# The Growth, Lipid Accumulation and Fatty Acid Profile Analysis by Abscisic Acid and Indol-3-Acetic Acid Induced in *Chlorella* sp. FACHB-8

**DOI:** 10.3390/ijms23074064

**Published:** 2022-04-06

**Authors:** Yihua Lin, Yue Dai, Weinan Xu, Xiaobin Wu, Yanyan Li, Hongmei Zhu, Hantao Zhou

**Affiliations:** 1 State Key Laboratory of Marine Environmental Science, Xiamen University, Xiamen 361102, China; linyihua@stu.xmu.edu.cn (Y.L.); xuweinan@stu.xmu.edu.cn (W.X.); wuxiaobin@stu.xmu.edu.cn (X.W.); yanyanli2016@stu.xmu.edu.cn (Y.L.); 2 College of Ocean and Earth Sciences, Xiamen University, Xiamen 361102, China; 22320182201300@stu.xmu.edu.cn (Y.D.); flyzhu324@163.com (H.Z.)

**Keywords:** plant hormones, fatty acid, microalgae, transcriptome

## Abstract

Microalgae are considered a promising source for biodiesel. The addition of plant hormone can exert a significant impact on the production of microalgae biomass and lipid accumulation. Nevertheless, the response of microalgae cells to hormones is species- or strain-dependent. It remains controversial which genes involved in strong increase of fatty acids production in response to abscisic acid (ABA) in *Chlorella* sp. FACHB-8 strain. We investigated cell growth, lipid accumulation, and fatty acid composition when ABA and indol-3-acetic acid (IAA) were used in the growth medium of *Chlorella* sp. FACHB-8. The four treatments, including 5 mg/L IAA (E1), 10 mg/L IAA (E2), 10 mg/L ABA (E3), the combination of 5 mg/L IAA and 5 mg/L ABA (E4), were found to increase cell growth, but only 10 mg/L ABA treatment could enhance the lipid accumulation. The fatty acid profile was changed by the addition of ABA, making fatty acids afflux from polyunsaturated fatty acids to monounsaturated and saturated fatty acids, which were suitable for diesel application. Furthermore, a transcriptome analysis was conducted, unraveling the differentially expressed genes enriched in fatty acid biosynthesis, fatty acid metabolism, and biosynthesis of the unsaturated fatty acid pathway in response to ABA. Our results clarified the correlation of fatty acid synthesis-related genes and fatty acid profiles, helping understand the potential response mechanism of *Chlorella* sp. FACHB-8 strain respond to ABA treatment.

## 1. Introduction

Microalgae have become one of the most promising and environmentally friendly sources of biodiesel production due to their rapid growth, oleaginicity, nonencroachment of arable land, and capability to grow in a wide range of waters [1]. Microalgae offer great potential for kinds of high-value products, including food and feed supplements, biofuels and bioactive compounds. When microalgae are cultured under unfavorable environmental conditions, algae can modify lipid metabolism and display unusual patterns of cellular lipids to respond to this change [2,3]. Microalgae shows the exploitable potential in their diverse fatty acid (FA) profile and abundance [4]. The variety of FAs in living organisms is primarily determined by differences in the length of the hydrocarbon chain, as well as by the number of double bonds [4]. In fact, different saturated FA (SFA; without double bond), monounsaturated FA (MUFA; with one double bond), and polyunsaturated FA (PUFA; with more than one double bond) were described in microalgae. Hexadecanoic acid (C16:0) and oleic acid (C18:1) are the common FAs observed among different microalgae, which are considered as preferred components for making biodiesel [5]. PUFAs especially omega-3 (ω-3) FA, have long been used as value-added nutraceuticals for the nervous system in humans [6,7]. FAs are involved in the metabolic pathway of formation and conversion of most lipid classes, and their composition largely determines their properties and practical use. Many factors, such as nitrogen deficiency [8,9], high salinity [10], and high temperature [11] have been shown to increase the oil content in microalgae, however, these methods decrease the microalgae growth. Lipid productivity takes into account both the lipid accumulation in the cells and the biomass produced [12]. Phytohormones provide a promising strategy to increase the lipid productivity due to their key role in microalgal growth, lipid accumulation and improving their tolerance to more extreme environmental changes [13].

Exogenous phytohormone application has been reported to enhance cell growth and high-value product accumulation in microalgae in previous study [14,15,16]. The addition of ABA, salicylic acid (SA), and jasmonates (JA) made two *Chlorella* strains produce a 1.8-, 1.7-, and 2.0-fold lipid content compared with the controls, respectively [17], which suggested that certain concentrations of these phytohormones play important roles in inducing lipid accumulation in *Chlorella*. Kozlova et al. showed that the combination of 24-epibrassinolide and IAA increased the biomass production, chlorophyll-a and carotenoid accumulation, and triacylglycerol (TAG) content of *S. quadricauda* [18]. It has also been reported that exogenous phytohormone treatments led to a significant increase in PUFAs in *Chlorella vulgaris*, which was confirmed by the upregulation of fatty acid biosynthesis genes [19]. The synergistic effect of combination of exogenous phytohormones in enhancing biomass and lipid productivity for microalgae biofuel production has been elucidated [20,21].

Although the physiological responses of microalgae to exogenous phytohormone application of *Chlorella* sp. has been investigated, what genes are involved in FA metabolism after ABA addition remain largely unknown. In this study, we firstly investigated the effects of IAA, ABA, and their mixtures on lipid accumulation, growth, and fatty acid profiles in the microalgae strain FACHB-8. ABA was thought to the hormone with an inductive effect on FA profile. To better understand the molecular mechanism behind this respond, the differential expression genes in the fatty acid metabolism pathway were analyzed through transcriptomic approach and verified by quantitative reverse transcriptase PCR (qPCR). These results can contribute to the understanding of the lipid accumulation and assignment in *Chlorella* sp. FACHB-8 in response to the ABA treatments, which may provide the target genes for genetic engineering in improving microalgae lipid productivity.

## 2. Results

### 2.1. The Taxonomic Assignment of FACHB-8

**Figure 1:**
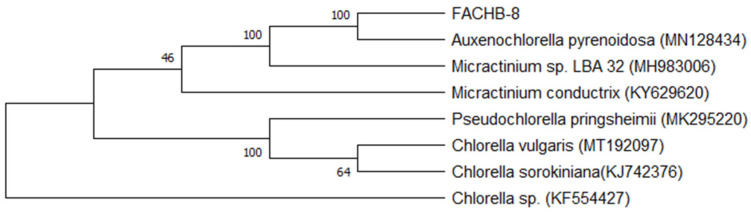
Phylogenetic tree of *rbcL* gene coding sequences with the neighbor-joining method. Numbers at the branches represent the percent of bootstraps supporting branches (*n* = 1000). GenBank number of sequences was included in the brackets.

The phylogenetic tree of rbcL transcripts was constructed by the neighbor-joining method, which showed that FACHB-8 had the highest similarity to *Auxenochlorella pyrenoidosa*, supported by 100% of the 1000 bootstraps (Figure 1).

### 2.2. The Hormone Effect on Growth in Chlorella sp. FACHB-8

**Figure 2:**
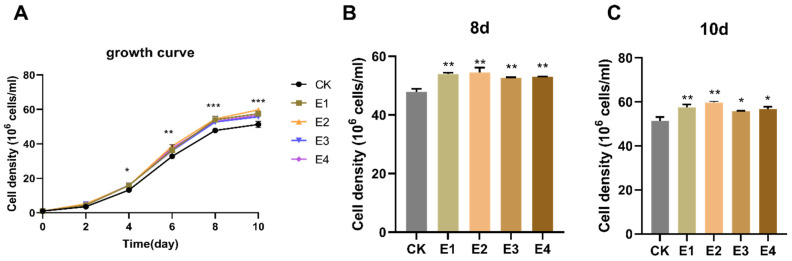
The growth curve and cell density of FACHB-8 with different supplemental hormone combinations. (**A**) Growth curve of FACHB-8 after inoculation. CK represents the control with an equal volume of ethanol. E1 (5 mg/L IAA), E2 (10 mg/L IAA), E3 (10 mg/L ABA), and E4 (5 mg/L IAA + 5 mg/L ABA) were also diluted with ethanol. The cell density of FACHB8 with different experimental treatments at (**B**) 8 days and (**C**) 10 days after inoculation. * means *p* < 0.05, ** indicated *p* < 0.01, *** means *p* < 0.001.

The plant hormones IAA and ABA and their combination were applied to FACHB-8 culture to further explore their potential inductive effects on growth. In this study, 5 mg/L IAA, 10 mg/L IAA, 10 mg/L ABA, and the combination of 5 mg/L IAA and 5 mg/L ABA were designed as experimental treatments, namely, E1, E2, E3, and E4, respectively. The IAA and ABA concentration used in this study were all proved to promote FACHB-8 cell growth during the logarithmic growth period and early stationary growth phase as shown in Figure 2A. The highest growth enhancement was found under the addition of 10 mg/L IAA, with a cell density of 5.4 × 10^7^ cells/mL, while the cell number of the control was 4.7 × 10^7^ cells/mL (Figure 2B). However, the hormone supplements increased the number of microalgae cells by 12.91%, 13.95%, 10.01%, and 10.96% compared with the control at 8 days after inoculation (Figure 2B). At 10 days, the enhancement percentages of growth were 12.19%, 16.55%, 8.55%, and 10.40%, respectively (Figure 2C). The results showed that the growth increase range of IAA supplementation in the microalgae FACHB-8 strain was greater than that of ABA addition.

### 2.3. The Hormone Effect on Carbohydrate, Protein, and Lipid Accumulation

**Figure 3:**
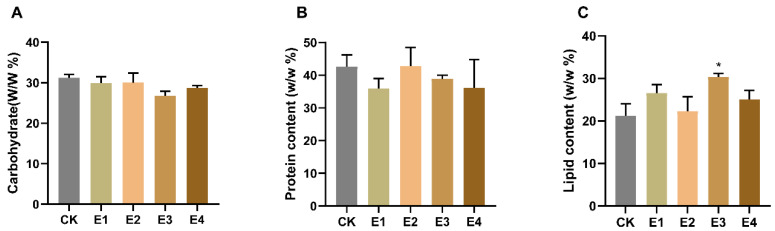
The content of (**A**) carbohydrate, (**B**) protein, and (**C**) lipid. CK represents the control, and E1-E4 represent 5 mg/L IAA, 10 mg/L IAA, 10 mg/L ABA, and 5 mg/L IAA + 5 mg/L ABA, respectively. * means *p* < 0.05.

Carbohydrate, protein, and lipid accumulation in response to plant hormones differed in the four plant hormone treatments. There was no significant variation (*p* > 0.05) in the carbohydrate and protein contents between the control and experimental groups (Figure 3A,B). However, the highest lipid content of 30.31% was produced by FACHB-8 under the treatment of 10 mg/L ABA (E3), whereas the total oil content of the control was only 21.23% (Figure 3C).

### 2.4. The Hormone Effect on Fatty Acid Composition

The fatty acid composition of microalgae was affected by the addition of auxin and abscisic acid. Under IAA doses of 5 mg/L and 10 mg/L (E1 and E2), the maximum C16:4 was found. However, SFA, MUFA, and PUFA contents of E1 and E2 were not significantly different from those of the control. The SFA content increased as the PUFA content declined significantly under the combination addition of 5 mg/L IAA and 5 mg/L ABA (Table 1, E4). The highest enhancement of SFAs and MUFAs was obtained at 10 mg/L ABA, with 27.37% and 41.58% enhancement compared with the control, respectively (Table 1, E3). The elevated accumulation of SFAs in ABA-treated cultures was mainly contributed by the increase in C16:0 (*p* < 0.05) content. However, the accumulation of MUFAs was increased to 6.77% when treated with 10 mg/L ABA compared to the control (4.78%), while the contents of palmitoleic acid (C16:1) and oleic acid (C18:1) were enhanced with nonsignificant differences (*p* > 0.05).

The PUFA content of E3 decreased with decreasing C16:4 and C18:3 contents. Interestingly, the combination of 5 mg/L IAA and 5 mg/L ABA treatment caused PUFA production to be lower than that of the control, which may be due to the contribution of ABA addition.

### 2.5. Enrichment and Identification of Differentially Expressed Genes

All six samples yielded 37.77 Gbp of high quality sequence from 260 million reads. This was assembled into a 57 Mbp transcriptome consisting of 33,314 transcripts and 25,047 genes, with an N50 value of 2772 bp (Supplementary Table S1). Benchmarking Using Single Copy Orthologs (BUSCO) v2.0 analysis [15] was used to assess the transcriptome completeness with a high map-back rate (93%), suggesting that the obtained sequencing data captured a wide representation of the expressed transcriptomes of the samples (Supplementary Figure S1). The raw read data were uploaded to NCBI, and the BioSample accessions of six samples were SAMN21595570-SAMN21595575 (shown in Supplementary Table S1).

**Figure 4:**
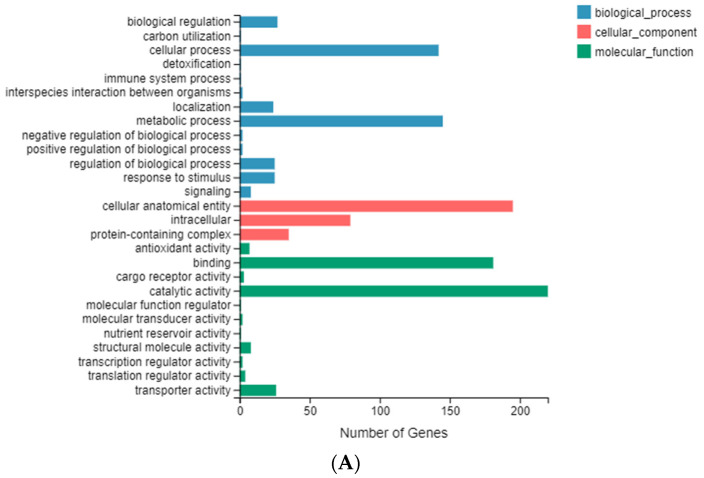

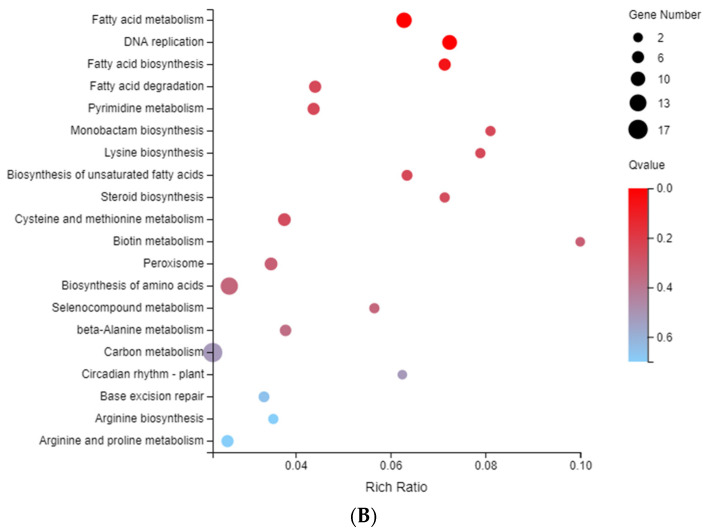
Pathways enriched under the (**A**) GO and (**B**) KEGG databases.

A total of 27,863 transcripts (83.64%) were assigned to either the KEGG, GO, NR, NT, SwissProt, Pfam, or KOG databases. A total of 9674 transcripts (29.04%) were annotated by all seven databases (Supplementary Table S2). Only 356 genes were considered to be differentially expressed, with 171 genes significantly upregulated, as determined using DESeq2 (FDR < 0.05). According to GO annotation results and classification, the differentially expressed genes were functionally classified into biological process, cellular component, and molecular function. Cellular processes and metabolic processes were the main parts of the biological process category. The cellular component consists of cellular anatomical entities and intracellular and protein-containing complexes. Binding and catalytic activity played an important role in the molecular function (Figure 4A). KEGG annotation results and official classification were used to classify the differential expressed genes into biological pathways. The pathways of fatty acid metabolism (11 genes), DNA replication (10 genes), fatty acid biosynthesis (six genes), and fatty acid degradation (six genes) were enriched (Figure 4B).

**Figure 5:**
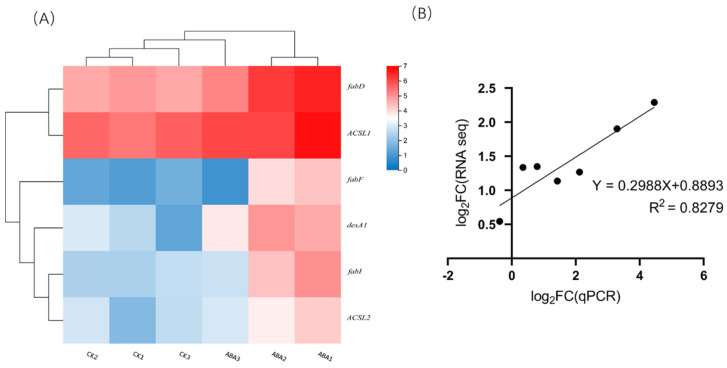
The gene cluster of Fatty acid biosynthesis pathway based on FPKM value (**A**) and the correlation analysis of RNA seq and qPCR data (**B**).

A total of nine genes were assigned to lipid metabolism, which belonged to the fatty acid metabolism pathway. Furthermore, six genes were found to participate in fatty acid elongation and desaturation. The gene cluster of ACP-S-malonyltransferase (*FabD*), 3-oxoacyl-ACP synthase II (*FabF*), enoyl-ACP reductase I (*FabI*), acyl-ACP desaturase (*DesA1*), and two long-chain acyl-CoA synthetase (*ACSL1* and *ACSL2*) was shown in Figure 5A and classified as fatty acid biosynthesis pathway. RT–qPCR was used to verify the relative expression of 7 gene transcripts, including *FabD*, *FabF*, *FabI*, *DesA1*, stearoyl-CoA desaturase (Delta-9 desaturase, *DesC*), *ASCL2* and 3-ketoacyl-CoA synthase. Seven genes were selected for correlation analysis. The log2 expression values of these seven genes are shown in Figure 5B, which means the qPCR data of these genes were consistent with the RNA-Seq results (Figure 5B).

**Figure 6:**
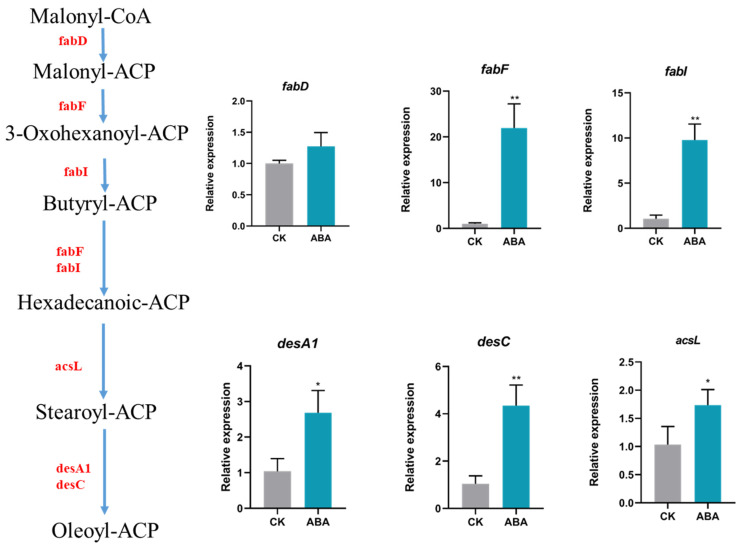
Pathway diagram and gene expression of reconstructed fatty acid-related processes, including fatty acid biosynthesis and desaturation for FACHB-8 under ABA treatment. Red font represents the upregulated genes in RNA-Seq, and the histogram describes the relative expression of fatty acid-related genes by RT–qPCR. *FabD*, ACP-S-malonyltransferase; *FabF*, 3-oxoacyl-ACP synthase II; *FabI*, enoyl-ACP reductase I; *DesA1*, acyl-ACP desaturase; *DesC*, stearoyl-CoA desaturase (Delta-9 desaturase); *AcsL*, long-chain acyl-CoA synthetase. CK means untreated cell and ABA indicates 10 mg/L ABA treatment. * means *p* < 0.05, ** indicated *p* < 0.01.

All six transcripts were significantly upregulated in the treatment group supplemented with 10 mg/L ABA. The six genes *FabD, fabF, fabI, desA1, desC, and acsL2* were upregulated 1.27-, 21.93-, 9.79-, 2.68-, 4.35-, and 1.73-fold, respectively (Figure 6). The gene encoded for *FabD* and *FabF* representing two key enzymes in the fatty acid synthesis pathway. *FabF* is implicated in transferase activity (transferring acyl groups other than amino-acyl groups).

## 3. Discussion

Phytohormones regulate the growth and development of plant and algae as well as boost tolerance to harsh environmental conditions [22]. Endogenous phytohormone levels fluctuate with growth and life cycle stages in microalgae, providing evidence that specific phytohormones regulate the cell cycle and hence growth rates. The faster growing species had a higher endogenous auxin concentration compared to the slower growing species in previous report, which suggests that auxins may play a role in cell division and growth in microalgae [23]. ABA is a stress regulator in plant cells, and it induces antioxidant enzyme activity to tolerate the biotic and abiotic stress in green microalgae and plants [24,25]. Exogenous phytohormones have the potential to increase microalgae productivity with eliciting multiple physiological responses [13]. Exogenous auxins (e.g., IAA and 2,4-dichlorophenoxyacetic acid) have been reported to promote cell growth in many microalgae species [16,20,26]. ABA treatment enhanced lipid productivity without hindering growth in *Chlorella* [17] and the oleaginous marine diatom [27]. In this study, 5 mg/L IAA, 10 mg/L IAA, 10 mg/L ABA and a combination of IAA and ABA (each 5 mg/L) were found to promote the FACHB-8 cell growth. The effects of IAA and ABA concentrations on microalgae cell growth are controversial [28,29,30]. This may be caused by the differences in microalgae species, strains, and culture conditions, including light intensity, light period, and medium element concentrations. ABA treatments caused an increase in cell number, which has been reported in the microalgae *C. saccharophila* [31]; however, ABA addition was shown to inhibit cell division in plants and decrease the growth of *Nannochloropsis oceanica* [15,32]. We also showed that the cell growth enhancement of IAA supplementation in the culture was greater than the equal amount of ABA addition in the FACHB-8 strain.

On the other hand, 10 mg/L ABA addition increased the lipid content of dry weight in the present study, which had the same trends as previous reports [18,28]. However, IAA-treated cells showed a slight increase in lipid content that was nonsignificant (*p* > 0.05). A study by Sivaramakrishnan [33] reported that IAA treatment in *Chlorella* sp. showed 51% lipid content, increasing by 20% compared with the control. This suggested that the response of cells to IAA is species-dependent.

The addition of different plant hormones affected the alga differently in terms of fatty acid compositions [33]. The content of C16:4, an important component of polyunsaturated fatty acids, increased with the addition of IAA at 5 mg/L and 10 mg/L in the present study. The SFA and PUFA contents after 10 days of IAA treatment were unchanged. However, Jusoh et al. reported that PUFA production was increased to 146.81% at Day 10 after addition and suggested that IAA promotes PUFAs in the late stationary growth phase [34]. Furthermore, the addition of ABA to the growth media of FACHB-8 significantly decreased the C16:4 and α-linolenic acid contents and simultaneously increased the SFA and MUFA contents, thereby improving the quality of the feedstock for biodiesel production. FA profiles are determined by the stain as well as culture conditions. A previous study of ABA treatment of *C. pyrenoidosa* showed a decreased PUFA content and reduced fatty acid desaturase expression [35]. The fatty acid with the longest carbon chain length appearing in *C. pyrenoidosa* was C20:4, whereas C18:3 was found in *Chlorella* sp. FACHB-8. Auxin treatments altered the TAG content along with an increase in the MUFA and a decrease in the PUFA content [26]. The treatments of IAA and diethyl aminoethyl hexanoate (DAH) significantly increased the ratios of PUFAs and decreased the SFAs in a dose-dependent manner [36,37]. FACHB-8 can synthesize C16:4 and C18:3, which also be reported in *C. reinhardtii* whereas they are not present in Arabidopsis. C16:0 and C18:1 can be either incorporated into the chloroplast membrane lipids or exported out of the chloroplast and then incorporated into the endoplasmic reticulum (ER) membrane lipids for further desaturation [38]. In addition, the content of C16:0 and C18:1 with ABA treatment was higher than untreated cells.

RNA-seq analysis helps to reveal the molecular mechanism underlying the significant improvement of cell growth and lipid content after the addition of ABA. *FabF* and *fabI* function in the elongation cycle as beta-ketoacyl synthase and enyol-ACP reductase, respectively. *DesA1* and *desC*, which function to position a single double bond into an acyl-ACP substrate, are fatty acid Delta-9 desaturases. The genes were upregulated due to ABA supplementation, leading to the contents of SFAs and MUFAs being higher than those of the control (Table 1). A significant correlation analysis between the gene expression of ACP, 3-ketoacyl-ACP-synthase, desaturase, and MUFA and PUFA synthesis was verified [39]. The results showed that the FPKM values of biosynthetic amino acid genes were not significantly different between ABA treatment and control, resulting in no change in protein content (Supplementary Figure S2 and Figure 3B). It has been reported that the transcription of fatty acid synthesis-related genes is upregulated after ABA treatment [14,31]. The study also opens up new targets for genetic engineering in enhancing the lipid productivity. 

Furthermore, Sivaramakrishnan et al. reported that plant hormones could stimulate fatty acid desaturase and elongation through oxidative stress. It is suggested that the addition of ABA makes *Chlorella* sp. fight against Reaction oxygen species (ROS) molecules by improving antioxidant enzyme levels [33]. Exogenous hormones induce the activities of superoxide dismutase (SOD) and catalase (CAT), which are important antioxidant enzymes that protect the photosynthetic apparatus and chloroplasts [40,41]. Indeed, the coding gene of peroxidase activity (peroxiredoxin (alkyl hydroperoxide reductase subunit C) [EC:1.11.1.15]) and ATP-dependent RNA helicase DOB1 [EC:3.6.4.13] were upregulated with 1.82- and 5.2-fold in our transcriptome data (Supplementary Table S3). Hence, plant hormone addition made algae cells fight against ROS-induced oxidative stress, which improved biomass and lipid content as well as upregulated fatty acid synthesis genes. ABA, as one of the phytohormone alternatives, indicated large possibilities in developing affordable and scalable microalgal lipids and is utilized for biodiesel applications with high contents of SFAs and biomass.

## 4. Materials and Methods

### 4.1. Microalgae Strain and Culture Conditions

The *Chlorella* sp. strain of FACHB-8 was purchased from the Freshwater Algae Culture Collection at the Institute of Hydrobiology, Chinese Academy of Sciences (Wuhan, China).

The strain was cultured in 75 cm^2^ tissue culture flasks with 50 mL BG11 medium [42] under illumination of 50 μmol m^−2^ s^−1^ with a 12 h light:12 h dark photoperiod at 22 °C. The culture was maintained and grown for 10 days and harvested for further investigations.

### 4.2. Hormone Treatments

IAA and ABA were purchased from LABLEAD Inc. (Beijing, China). Four experiments, including 5 mg/L IAA (E1), 10 mg/L IAA (E2), 10 mg/L ABA (E3), and 5 mg/L IAA plus 5 mg/L ABA (E4) were added to the cultures, which was carried out in 50 mL BG11 medium, while an equal volume of ethanol was grown as a control (CK) because the hormones were dissolved in 100% ethanol. Each treatment was conducted by three repeats. The cell number was detected every two days, and cells were harvested at day 10 after being treated by centrifugation with washing twice in Milli-Q water. The harvested cells were freeze-dried and stored at −80 °C until protein, carbohydrate, lipid and fatty acid extraction took place.

### 4.3. Analyses of Microalgae Growth, Protein and Carbohydrate Contents

The cell concentration was determined by flow cytometry CytoFLEX S (Beckman, IN, USA). The protein and carbohydrate contents were determined by a Bradford Protein Assay Kit and Total Sugar Content Detection Kit (Solarbio, Beijing, China). All data were analyzed with one-way ANOVA (GraphPad Prism 8). Three experimental repeats were conducted.

### 4.4. Total Lipid and Fatty Acid Profile Determination

Frozen microalgae powder (20 mg) was ground with liquid nitrogen and dissolved in chloroform: methanol (*v*/*v*, 1:2). After centrifugation, the supernatant was collected into a new tube, dried with nitrogen, and then weighed. The dried extraction was re-dissolved in 1 mL of CH_3_OH solution, which contained 1% H_2_SO_4_ for methyl esterification. After filling the tube with nitrogen, the mixture was vortexed and incubated in a water bath for 60 min at 80 °C. The phase containing the fatty acids was obtained using 2 mL of hexane. After standing and layering, the fatty acid phase was transferred into a new glass tube with 10 ppm C19:0 and subjected to GC-MS analysis [43]. The gas chromatography-mass spectrometry column was a CP-Wax-52 CB column (30 m × 0.25 mm × 0.25 μm) with the injection temperature was 230 °C. The head temperature was 50 °C, and lasted for 5 min, then the temperature increased at 170 °C for 10 min, further increased to 205 °C/min at a rate of 3 °C /min with persisting for 25 min, and finally heated to 210 °C for 2 min at 5 °C/min. The reference standard Supelco 37 Component FAME Mix (Sigma Aldrich, Shanghai, China) was used to identify the fatty acids by comparing the peaks and retention times. Each sample included four biological replicates.

### 4.5. RNA-Seq

The treated cells with the greater cell number and lipid content (10 mg/L ABA, *n* = 3) and the untreated cells (CK, *n* = 3) were collected at the log phase (3 days), and then the cell pellets were immediately flash frozen with liquid nitrogen. Total RNA was extracted from the samples using the Polysaccharide/polyphenol Plant Total RNA Mini Kit (GeneBetter^®^, Beijing, China) according to the manufacturer’s instructions. Subsequently, total RNA was qualified and quantified using a NanoDrop and Agilent 2100 bioanalyzer (Thermo Fisher Scientific, Shanghai, China).

mRNA was enriched by binding to oligo (dT)-attached magnetic beads and fragmented into small pieces with fragment buffer at the appropriate temperature. Afterward, random N6 primers were used in reverse transcription to generate first-strand cDNA, followed by second-strand cDNA synthesis. The synthesized cDNA was subjected to end repair and then 3’ adenylated. Adapters were ligated to the ends of these 3’ adenylated cDNA fragments. The cDNA fragments were amplified by PCR and further validated for quality control. The PCR products from the previous step were heated, denatured, and circularized by the splint oligo sequence to obtain the final library. DNA nanoballs (DNBs), which were amplified from the final library, were loaded into the patterned nanoarray. Single-end 50-base reads were generated on the BGISeq500 platform (BGI-Shenzhen, China).

### 4.6. De Novo Assembly and Transcriptome Analysis

Raw reads were filtered via SOAP nuke (v1.4.0) [44], and then the obtained clean reads were assembled by Trinity (v2.0.6) [45]. Tgicl (v2.0.3) [46] was used to perform clustering and eliminate redundant data in the assembled transcripts to obtain unique genes. Furthermore, the assembled transcripts were processed for further expression analysis and functional annotation. Clean reads were mapped to the unique gene by Bowtie2 [47], and the gene expression was calculated by RSEM [48] and normalized to FPKM. Different databases, including NT, NR, KOG, and KEGG, were used for functional annotation of the genes achieved by BLAST (v2.2.23) [49]. However, GO annotation was conducted by Blast2GO (v2.5.0). Differentially expressed genes (DEGs) were detected by DEseq2 [50] with an absolute fold change value greater than 2 and an adjusted *p* value ≤ 0.001. Subsequently, GO and KEGG enrichment analyses were performed using Phyper (a function of R) with the significant levels of pathways and terms corrected by Q value < 0.05.

### 4.7. Taxonomic Assignment of FACHB-8 Strain

The coding sequences of the *rbcL* gene were used to identify the taxonomic assignment of FACHB-8 at the species level. The *rbcL* sequence was derived from the transcriptome and blasted in the GenBank database. The sequences of *Auxenochlorella pyrenoidosa* (MN128434), *Micractinium* sp. LBA 32 (MH983006), *Micractinium conductrix* (KY629620), *Pseudochlorella pringsheimii* (MK295220), *Chlorella vulgaris* (MT192097), *Chlorella sorokiniana* (KJ742376), and *Chlorella* sp. (KF554427) were downloaded from GenBank with high similarity. A total of eight sequences were aligned with MUSCLE, and a phylogenetic tree was constructed with MEGA X [51] using the neighbor-joining method and 1000 bootstrap replicates.

### 4.8. qRT–PCR Confirmation

cDNA was synthesized using an Evo M-MLV RT Kit with gDNA Clean for qPCR (Accurate Biotechnology Co., Ltd., Changsha, China) from 10 ng RNA. qPCR was carried out by a SYBR^®^ Green Premix Pro Taq HS qPCR Kit (Accurate Biotechnology Co., Ltd., Changsha, China) following the manufacturer’s instructions. A total of 20 µL reaction volume contained 2 µL cDNA, 10 µL SYBR Green (ROX mixed), and 0.4 µL 10 µM forward and reverse primers. The PCR programs were initiated at 95 °C for 20 s, followed by 40 cycles of 95 °C for 5 s and 60 °C for 30 s. The primers were screened by melt curve analysis with *tubulin* as the reference gene. The mRNA levels were quantified using the double delta Ct method [52]. The primer sequences are presented in Supplementary Table S4. All experiments were performed with at least three biological repeats; within each, three technical repeats were generated.

## 5. Conclusions

All the concentrations of IAA and ABA used in this study could promote the microalgae cell growth, but only 10 mg/L ABA treatment could enhance the lipid accumulation. The fatty acid profile was changed by the addition of ABA, making fatty acids afflux from polyunsaturated fatty acids to monounsaturated and saturated fatty acids, which can help improve the quality of the biodiesel feedstock. Fatty acid synthesis-related genes, including *FabD, fabF, fabI, desA1, desC,* and *acsL*, were screened and verified at the transcriptional level, which may offer the targets for genetic modification in enhancing microalgae biomass and customizing the fatty acid profile.

## Figures and Tables

**Table 1:** Fatty acid composition of microalgae FACHB-8 strain under hormone treatment.

Treatments	CK	E1	E2	E3	E4
C14:0 (%)	1.51 ± 0.26	1.60 ± 0.05	1.34 ± 0.08	1.93 ± 0.37	1.61 ± 0.31
C15:0 (%)	1.08 ± 0.21	1.13 ± 0.02	0.96 ± 0.04	1.39 ± 0.26	1.15 ± 0.23
C16:0 (%)	18.74 ± 1.17	18.65 ± 0.06	18.72 ± 0.42	23.85 ± 1.26 ***	20.21 ± 1.05
C18:0 (%)	2.43 ± 0.04	2.18 ± 0.14	1.68 ± 0.08	3.12 ± 0.54	2.49 ± 0.18
**SFA (%)**	**23.78 ± 0.66**	**23.56 ± 0.004**	**22.69 ± 0.23**	30.28 ± 0.09 ***	25.46 ± 0.33 *
C16:1 (%)	2.92 ± 0.67	2.95 ± 0.72	3.54 ± 0.14	4.14 ± 0.08	3.61 ± 0.03
C18:1N9C (%)	1.86 ± 0.15	1.79 ± 0.02	1.80 ± 0.41	2.63 ± 0.47	2.10 ± 0.58
**MUFA (%)**	**4.78 ± 0.52**	**4.74 ± 0.70**	**5.34 ± 0.55**	6.77 ± 0.39 **	**5.71 ± 0.61**
C16:2 (%)	3.32 ± 0.03	3.04 ± 0.09	2.25 ± 0.84	3.62 ± 0.15	2.56 ± 0.80
C16:3 (%)	11.94 ± 0.27	10.28 ± 0.28	10.59 ± 0.31	10.74 ± 0.86	10.19 ± 0.36
C16:4 (%)	11.21 ± 1.04	13.61 ± 0.33 **	13.95 ± 0.17 **	6.73 ± 3.44 ***	12.38 ± 1.00
C18:2n6c (%)	11.86 ± 0.92	10.56 ± 0.33	10.40 ± 0.13	12.51 ± 1.19	11.10 ± 0.65
C18:3n3 (%)	33.12 ± 0.82	34.22 ± 0.32	34.78 ± 0.10	29.35 ± 1.54 ***	32.60 ± 0.19
**PUFA (%)**	**71.44 ± 1.19**	**71.70 ± 0.70**	**71.97 ± 0.32**	62.95 ± 0.30 ***	68.83 ± 0.28 ***
**UFA (%)**	**76.22 ± 0.66**	**76.44 ± 0.004**	**77.31 ± 0.22**	69.72 ± 0.09 ***	74.54 ± 0.33 *

The numbers are avg. + se with *n* = 4 separately grown biomass. CK represents the control, and E1-E4 represent 5 mg/L IAA, 10 mg/L IAA, 10 mg/L ABA and 5 mg/L IAA + 5 mg/L ABA, respectively. The asterisks indicate statistically significant differences compared to controls. * means *p* < 0.05, ** indicated *p* < 0.01, *** means *p* <0.001.

## Data Availability

The RNA sequencing read data were deposited in the GenBank SRA database under the accession number PRJNA765954.

## Supplementary Materials

The following supporting information can be downloaded at: https://www.mdpi.com/article/10.3390/ijms23074064/s1.
