# Supplementary figures and images for: The Growth, Lipid Accumulation and Fatty Acid Profile Analysis by Abscisic Acid and Indol-3-Acetic Acid Induced in Chlorella sp. FACHB-8
Source: Int J Mol Sci. 2022 Apr 6;23(7):4064. doi: 10.3390/ijms23074064 (PMC8999460; doi:10.3390/ijms23074064)

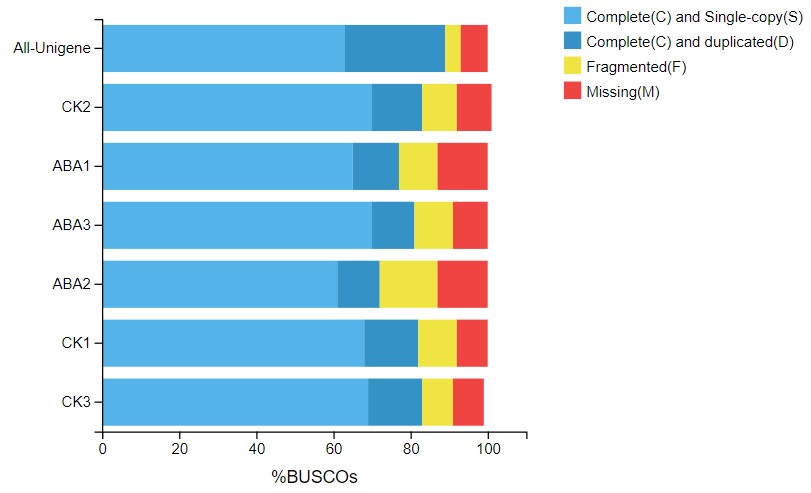

Supplement: Supplementary file 1 [file ijms-23-04064-s001.zip › Figure S1.jpg]

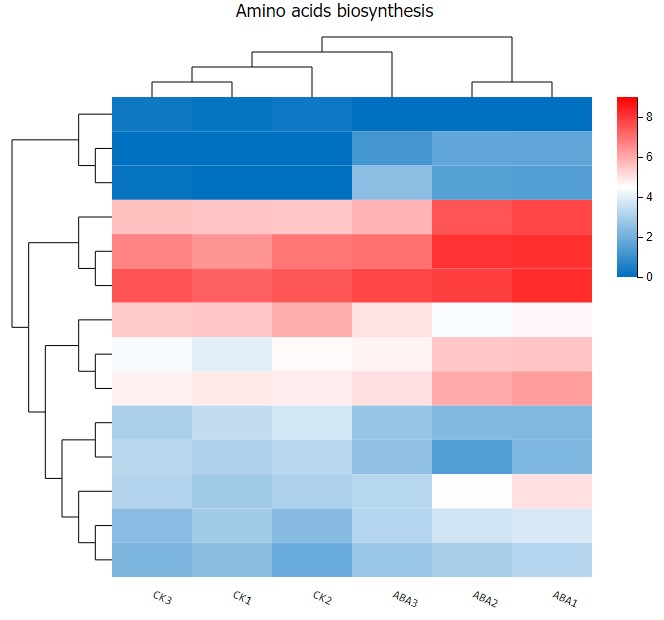

Supplement: Supplementary file 1 [file ijms-23-04064-s001.zip › Figure S2.jpg]
